# Intrarenal arterial administration of human umbilical cord-derived mesenchymal stem cells effectively preserved the residual renal function of diabetic kidney disease in rat

**DOI:** 10.1186/s13287-022-02857-5

**Published:** 2022-05-07

**Authors:** Ya Yue, Jui-Ning Yeh, John Y. Chiang, Pei-Hsun Sung, Yi-Ling Chen, Fanna Liu, Hon-Kan Yip

**Affiliations:** 1 Institute of Nephrology and Blood Purification, The First Affiliated Hospital of Jinan University, Jinan University, Guangzhou, 510632 China; 2 Department of Cardiology, The First Affiliated Hospital, Jinan University, Guangzhou, 510632 China; 3 Department of Computer Science and Engineering, National Sun Yat-Sen University, Kaohsiung, 804201 Taiwan; 4 Department of Healthcare Administration and Medical Informatics, Kaohsiung Medical University, Kaohsiung, 80708 Taiwan; 5 Division of Cardiology, Department of Internal Medicine, Kaohsiung Chang Gung Memorial Hospital and Chang Gung University College of Medicine, Kaohsiung, 83301 Taiwan; 6 Institute for Translational Research in Biomedicine, Kaohsiung Chang Gung Memorial Hospital, Kaohsiung, 83301 Taiwan; 7 Center for Shockwave Medicine and Tissue Engineering, Kaohsiung Chang Gung Memorial Hospital, Kaohsiung, 83301 Taiwan; 8 Department of Medical Research, China Medical University Hospital, China Medical University, Taichung, 40402 Taiwan; 9 Department of Nursing, Asia University, Taichung, 41354 Taiwan; 10 Division of Cardiology, Department of Internal Medicine, Xiamen Chang Gung Hospital, Xiamen, 361028 Fujian China

**Keywords:** Diabetic CKD, Renal function, Xenogeneic mesenchymal stem cell

## Abstract

**Background:**

This experimental study was designed as a preclinical study for testing the hypothesis that intrarenal arterial (IRA) transfusion of human umbilical cord-derived mesenchymal stem cells (HUCDMSCs) therapy preserved the residual renal function of diabetic kidney disease (DKD) in rat [induction by 5/6 nephrectomy of left kidney and right nephrectomy, followed by intraperitoneal administration of aminoguanidine (180 mg/kg) and streptozotocin (30 mg/kg)].

**Methods:**

Animals (*n* = 24) were categorized into group 1 (sham-operated control), group 2 (DKD), group 3 [DKD + HUCDMSCs (2.1 × 10^5^/IRA injection at day 28 after CKD induction)] and group 4 [(DKD + HUCDMSCs (6.3 × 10^5^/IRA injection)].

**Results:**

By day 60 after DKD induction, the kidneys were harvested and the result showed that the creatinine level, ratio of urine protein/urine creatinine and kidney injury score were lowest in group 1, highest in group 2 and significantly lower in group 4 than in group 3 (all *p* < 0.0001). The protein expressions of apoptotic (cleaved caspase-3/cleaved PARP/mitochondrial Bax), fibrotic (TGF-ß/p-Smad3), autophagic (ratio of LC3B-II/LC3B-I, Atg5/Beclin-1), oxidative stress (NOX-1/NOX-2/oxidized protein/p22phox), mitochondrial/DNA-damaged (cytosolic-cytochrome-C/DRP1**/**γ-H2AX) and inflammatory (MMP-9/TNF-α/p-NF-κB) biomarkers exhibited an identical pattern, whereas the protein expressions of angiogenesis factors (CD31/vWF/vascularity) exhibited an opposite pattern of creatinine level among the groups (all *p* < 0.0001). Histopathological findings demonstrated the renal tubular-damaged (KIM-1)/kidney fibrosis area/oxidative stress (8-OHdG + cells) expressed an identical pattern, whereas the podocyte components (ZO-1/synaptopodin/podocin) exhibited an opposite pattern of creatinine level among the groups (all *p* < 0.0001). No tumorigenesis or immune rejection event was identified.

**Conclusion:**

IRA injection of xenogeneic MSCs was safe and effectively protected the residual renal function and architectural integrity in DKD rat.

## Introduction

Plentiful data from clinical observational studies have clearly revealed that CKD contributed high morbidity and mortality in hospitalized patients, especially in those CKD patients with coexisting cardiovascular disease (i.e., cardiorenal syndrome) [1–6]. Surprisingly, despite the state-of-the-art therapeutic and advanced pharmaceutical strategies, such as the uses of angiotensin-converting enzyme inhibitor (ACEI), angiotensin II type I receptor blockade (ARB), direct renin inhibitor (DRI) and good education regarding how to avoid damage of kidney as well as renewed guidelines for CKD precise management, progressive deterioration of kidney function still inevitably occurs in a majority of CKD patients, subsequently progresses into end-stage renal disease (ESRD) in these patients [7–10]. Thereby, to find a new, effective and safe strategic management is not only of utmost importance for patients and physicians but also important to lower the medical cost in the world.

Through keen investigation lasted more than several decades, the etiology of CKD has been clearly shown to be divergent and the mechanisms involved are complicated [11–15]. Additionally, numerous data have shown that diabetic nephropathy (DN) is a major serious complication of diabetes and is also the most common cause of ESRD with poor prognosis and high cost for therapy [16–19]. Study has further identified that an early sign of DN is an increased protein release in urine, displayed as microalbuminuria, which is associated with the progression of renal damage, including glomerular hypertrophy, hyperfiltration, widening of basement membranes, tubule-interstitial fibrosis, glomerulosclerosis and podocytopathy [20]. In particular, macrophage infiltration, inflammatory reactions, TGF-ß expression, fibrosis formation and generations of oxidative stress and reactive oxygen species (ROS) have been reported as the principal mechanisms involved in the CKD [21–29]. Taken into consideration of various etiologies and the intricate mechanisms involved [21–29], resorting only to conventional therapies for prevention of CKD development into ESRD would be a mission impossible. Therefore, finding an innovative therapeutic strategy with relatively broad-spectrum effect for preservation of renal microvasculature/endothelial function, glomerulus/basement membrane integrity and residual renal function is of fundamental importance.

Recently, we have conducted a phase I [30] and a phase II [31] clinical trials of autologous CD34 + cell transfusion into the intrarenal arteries of stage 3–4 CKD patients, respectively. Although the safety of CD34 + cell therapy for the CKD patients has been proved, the efficacy for improvement in the renal function was not observed by these two clinical trials [30, 31], implicating that CD34 + cells and other endothelial progenitor cells (EPCs) may not have the reliable ability to overcome the intricate mechanisms that participate in CKD initiation and propagation [21–29].

Abundant data have shown that mesenchymal stem cells (MSCs) therapy effectively protected the organs from different disease entities mainly through inhibiting the inflammation and innate and adaptive immunity through down-regulating immunogenicity [32–37]. Our recent phase I clinical trial also demonstrated that human umbilical cord-derived mesenchymal stem cells (HUCDMSCs) therapy was safe and life-saving for moderate–severe acute respiratory distress syndrome (ARDS) patients [38]. However, various aspects of MSC therapy, especially when intrarenal arterial transfusion of HUCDMSCs is considered, have not yet been reported. We are now in the process of preparing IRB documents for a phase I clinical trial of HUCDMSCs therapy for stage 3–4 diabetic CKD (i.e., DKD) patients. However, prior to carrying out this clinical trial, a preclinical trial, i.e., an animal model of DKD with HUCDMSCs therapy for testing the safety and efficacy has been requested by Taiwan FDA (i.e., abbreviated as TFDA). This was the main reason for performing this preclinical animal study.

## Materials and methods

### Ethics

All animal procedures were approved by the Institute of Animal Care and Use Committee at Kaohsiung Chang Gung Memorial Hospital (Affidavit of Approval of Animal Use Protocol No. 2018102602) and performed in accordance with the Guide for the Care and Use of Laboratory Animals.

Animals were housed in an Association for Assessment and Accreditation of Laboratory Animal Care International (AAALAC; Frederick, MD, USA)-approved animal facility in our hospital with controlled temperature and light cycles (24 °C and 12/12 light cycle).

### Animal model of CKD induction, animal grouping, diabetic CKD (DKD) induction, blood sugar monitor and definition of diabetes mellitus

Pathogen-free, adult male Sprague Dawley (SD) rats (*n* = 24) weighing 320–350 g (Charles River Technology, BioLASCO Taiwan Co. Ltd., Taiwan) were utilized in the current study. The procedure and protocol of CKD induction have been described in our previous study [35]. In detail, all animals were anesthetized by inhalational 2.0% isoflurane and placed supine on a warming pad at 37 °C for midline laparotomies. The sham-operated control (SC) rats received laparotomy only, while CKD was induced in all animals of the CKD groups by right nephrectomy plus arterial ligation of the upper two-third (upper and middle poles) blood supplies of the left kidney, leaving the lower third (lower pole) kidney with normal blood supply. This model allowed preservation of a limited amount of functioning renal parenchyma and simulation of CKD.

The diabetic induction was performed at day 2 after CKD induction. The procedure and protocol of diabetes mellitus (DM) induction were based on our previous report [39] with some modification. In detail, streptozotocin (STZ) (30 mg/kg) and aminoguanidine (180 mg/kg) [40] [i.e., for protecting the beta cells (β-cells) in islets of pancreas from being completely destroyed, equivalent to a model of type 2 DM] were intraperitoneally administered by day 7 after the complete procedure of CKD induction was performed one time only, resulting in a DKD animal model. Additionally, the animal grouping, i.e., categorized the DKD animals into without and with HUCDMSCs treatment, was performed by day 14 after CKD induction (i.e., by day 7 after DKD induction). The rationale and dosage of aminoguanidine utilized in the present study were based on the previous report [40], because this study [40] demonstrated that aminoguanidine could stimulate the insulin release and biosynthesis and inhibit the advanced glycosylation end-products (AGEs) formation and accumulation in islets, resulting in reducing the glucotoxicity toward β-cells.

Animals were then categorized into group 1 (SC), group 2 (DKD), group 3 [DKD + HUCDMSCs (2.1 × 10^5^ by intrarenal arterial injection at day 21 after CKD induction)] and group 4 [DKD + HUCDMSCs (6.3 × 10^5^ by intrarenal arterial injection at day 21 after CKD induction)]. The dosage of the HUCDMSCs administration was based on our previous report [41] with minimal modification.

By day 21 after CKD induction (refer to Fig. 10), the laparotomy was performed again, and the renal artery was identified by visual observation. Next, the HUCDMSCs inside a 30# needle were carefully and slowly injected into the renal artery by a senior technician who is an expert of CKD animal study. After the transfusion, the needle was removed and gentle compression was performed to the needle wound of renal artery until the bleeding was stopped, i.e., usually takes one minute.

The procedure and protocol for monitoring the circulating level of blood sugar and definition of diabetes mellitus were based on our previous report [38]. Briefly, the blood glucose level of each rat was examined at 8:00–9:00 a.m. using a blood glucose monitor (ACCU-CHEK-Active; Roche) by days 7 (i.e., at day 14 after CKD induction) and 60 after DKD induction. By day 7 after STZ treatment, a blood glucose level ≥ 250 mg/dL was defined as diabetes mellitus.

### The procedure and protocol for post-surgery monitoring and pain relief of the animals and animal euthanasia

After the CKD induction procedure, the animals were then allowed to recover in a warmed cage with free access to food and water. The recovery period was about 30 min. Finally, the animals were cared for in a portable animal intensive care unit (ThermoCare**®**) with food and water for 24 h. For analgesia, buprenorphine (0.05 mg/kg) was provided subcutaneously every 12 h for 48 h post-CKD induction procedure.

Euthanasia of animals was performed under anesthesia with overdose of isoflurane inhalation (i.e., more than 5.0%), and a blood sample more than 10 ml was collected for each rat. The kidneys were harvested after the respiratory arrest.

### Assessment of blood urine nitrogen (BUN) and creatinine levels

To determine whether the animal model of CKD was successfully created and the impact of HUCDMSCs therapy on protecting the renal function, blood samples were serially collected before and after the CKD procedure (i.e., prior to and at days 28 and 60 before the animals were euthanized). Serum levels of creatinine and BUN were measured in duplicate using standard laboratory equipment.

### Collection of 24-h urine for the ratio of urine protein to creatinine at baseline and at days 28 and 60 after DKD induction

The procedure and protocol have been described in our previous report [42]. For the collection of 24-h urine in individual study, each animal was put into a metabolic cage [DXL-D, space: 190 × 290 × 550 mm^3^, Suzhou Fengshi Laboratory Animal Equipment Co. Ltd., China] for 24 h with free access to food and water. Urine in 24 h was collected in all animals prior to and at days 28 and 60 after CKD induction for determining the ratio of urine protein to urine creatinine.

### Histopathological assessment of kidney injury score at day 60 after DKD induction

The histopathological scoring of kidney injury was determined in a blinded fashion as we previously reported [42]. Briefly, the left kidney specimens from all animals were fixed in 10% buffered formalin, embedded in paraffin, sectioned at 4 µm and stained (hematoxylin and eosin; H & E) for light microscopy. The score reflected the grading of tubular necrosis, loss of brush border, cast formation and tubular dilatation in 10 randomly chosen, non-overlapping fields (200x) for each animal as follows: 0 (none), 1 (≤ 10%), 2 (11–25%), 3 (26–45%), 4 (46–75%) and 5 (≥ 76%).

### Western blot analysis of left kidney specimens

The procedure and protocol have been described in our previous reports [38, 39, 41, 42]. In detail, equal amounts (50 μg) of protein extracts were loaded and separated by SDS-PAGE using acrylamide gradients. After electrophoresis, the separated proteins were transferred electrophoretically to a polyvinylidene difluoride (PVDF) membrane (Amersham Biosciences). Nonspecific sites were blocked by incubation of the membrane in blocking buffer [5% nonfat dry milk in T-TBS (TBS containing 0.05% Tween 20)] overnight. The membranes were incubated with the indicated primary antibodies [mitochondrial Bax (mit-Bax) (1:1000, Abcam, ab32503, Cambridge, UK), cleaved caspase-3 (c-Csp3) (1:1000, Cell Signaling, #9662S, Danvers, MA, USA), cleaved poly (ADP-ribose) polymerase (c-PARP) (1:1000, Cell Signaling, #9542, Danvers, MA, USA), phosphorylated (p)-Smad3 (1:1000, Cell Signaling, #9520, Danvers, MA, USA), p-Smad1/5 (1:1000, Cell Signaling, #9516, Danvers, MA, USA), transforming growth factor (TGF)-β (1:500, Abcam, ab64715, Cambridge, UK), p22phox (1:1000, Abcam, ab191512, Cambridge, UK), endothelial nitric oxide synthase (eNOS) (1:1000, Abcam, ab76198, Cambridge, UK), von Willebrand factor (vWF) (1:1000, Abcam, ab174290, Cambridge, UK), matrix metalloproteinase (MMP)-9 (1:1000, Abcam, ab76003, Cambridge, UK), tumor necrosis factor (TNF)-α (1:1000, Cell Signaling, #3707, Danvers, MA, USA), phosphorylated nuclear factor (p-NF)-κB (1:1000, Cell Signaling, #3033, Danvers, MA, USA), Atg5 (1:1000, Cell Signaling, #12994, Danvers, MA, USA), Beclin1 (1:1000, Cell Signaling, #3495, Danvers, MA, USA), dynamin-related protein 1 (DRP1) (1:1000, Cell Signaling, #8570, Danvers, MA, USA), γ-H2AX (1:1000, Cell Signaling, #9718, Danvers, MA, USA), LC3BI/II (1:1000, Cell Signaling, #2775S, Danvers, MA, US), NOX-1 (1:1000, Sigma-Aldrich, SAB4200097, Burlington, MA, USA), NOX-2 (1:1000, Sigma-Aldrich, SAB4200118, Burlington, MA, USA), superoxide dismutase (SOD) (1:1000, Abcam, ab16831, Cambridge, UK), mitochondrial cytochrome C (mit-CytoC) (1:1000, BD Biosciences, 556433, Franklin Lakes, NJ, USA), cytosolic cytochrome C (cyt-CytoC) (1:1000, BD Biosciences, 556433, Franklin Lakes, NJ, USA), vascular endothelial growth factor (VEGF) (1:1000, Abcam, ab1316, Cambridge, UK), CD31 (1:1000, Abcam, ab24590, Cambridge, UK) and actin (1:10,000, Millipore, MAB1501, Burlington, MA, USA)] for 1 h at room temperature. Horseradish peroxidase-conjugated anti-rabbit immunoglobulin IgG (1:3000, Sigma-Aldrich, A0545, Burlington, MA, USA) was used as a secondary antibody for 1-hour incubation at room temperature. The washing procedure was repeated eight times within 1 hour. Immunoreactive bands were visualized by enhanced chemiluminescence (ECL; Amersham Biosciences, Amersham, UK) and exposed to BioMax L film (Kodak, Rochester, NY, USA). For the purpose of quantification, ECL signals were digitized using Labworks software (UVP, Waltham, MA, USA).

### Immunohistochemical (IHC) and immunofluorescent (IF) studies

The procedures and protocols for IHC and IF examinations were based on our previous reports [38, 39, 41, 42]. Briefly, IF staining was performed for the examinations of ZO-1 (1:200, Abcam, ab59720, Cambridge, UK), kidney injury molecule (KIM)-1 (1:400, Novus Biologicals, AF3689, Centennial, CO, USA), synaptopodin (1:500, Santa Cruz Biotechnology, Inc., sc-21537, Santa Cruz, CA, USA) and podocin (1:100, Abcam, ab93650, Cambridge, UK), whereas the IHC staining was performed for identification of podocin (1:100, Abcam, ab93650, Cambridge, UK), 8-hydroxy-2'-deoxyguanosine (8-OHdG) (1:500, Abcam, ab93650, Cambridge, UK) and α-SMA (1:400, Sigma-Aldrich, A2547, Burlington, MA, USA). Respective primary antibody was used with irrelevant antibodies as controls. Three sections of kidney specimens were analyzed in each rat. For quantification, three randomly selected HPFs of microscope (200 × for IHC and IF studies) were analyzed in each section. The mean number per HPF for each animal was determined by summation of all numbers divided by 9. Additionally, the tissue slides were interpreted by an expert of rodent pathology.

An IHC-based scoring system was adopted for semiquantitative analysis of podocin and 8-OHdG in the kidney as a percentage of positive cells in a blinded fashion [score of positively stained cell: 0 = negative staining; 1 = 1–15%; 2 = 16–25%; 3 = 26–50%; 4 = 51–75%; 5 = 76–100% per high-power field (HPF)]. Additionally, an IF-based scoring system was adopted for semiquantitative analysis of KIM-1 in the kidney that was identical to the method outlined above for the analysis of podocin.

Furthermore, the fluorescence intensities (optical densities) of ZO-1 and synaptopodin were examined by fluorescence microscope (Olympus BX51: fluorescence imaging system of OLYMPUS cellSens Standard 1.17) with digital microscope camera of Olympus DP80 and captured and converted to arbitrary units (AU) by ImageJ software 1.53 edition. Fluorescence quantification was utilized to confirm the area of integrated intensity and mean grey value to be selected. The fluorescence intensity indicated the integrated density, i.e., area x average fluorescence of background readings.

### The details of specificity of antibodies used in Western blotting and immunostaining (i.e., specificity verification and controls)

In this study, we performed control staining to ensure that the observed staining pattern was specific and authentic. We incubated the positive control tissue that was known to express the protein of interest with primary antibody. The positive control proved that the staining protocol and antibody worked as expected. On the other hand, we also incubated the tissue with isotype-specific immunoglobulins, i.e., negative control. This negative control ensured that no cross-reactions or non-specific background signals were observed thanks to the secondary antibody and the detection reagents. All antibodies for IHC, IF and western blot have been broadly listed and adopted in peer-reviewed journal articles.

### Statistical analysis

Quantitative data are expressed as mean ± SD. Statistical analyses were performed using SAS statistical software for Windows version 8.2 (SAS Institute, Cary, NC, USA). ANOVA was conducted followed by Bonferroni multiple comparison post hoc test for comparing variables among groups. A probability value < 0.05 was considered statistically significant.

## Results

### Animal death during DKD induction and time courses of circulating levels of BUN and creatinine and the ratio of urine protein to urine creatinine

After DKD induction prior to the grouping, 11 of 40 animals were dead (i.e., all deaths occurred within days 6–11 after CKD induction). Thus, the mortality rate was 27.5% during DKD induction. Additionally, after animal grouping till the end of the study period, animal deaths in groups 1 to 4 were 0, 2, 1 and 1, respectively.

**Fig. 1:**
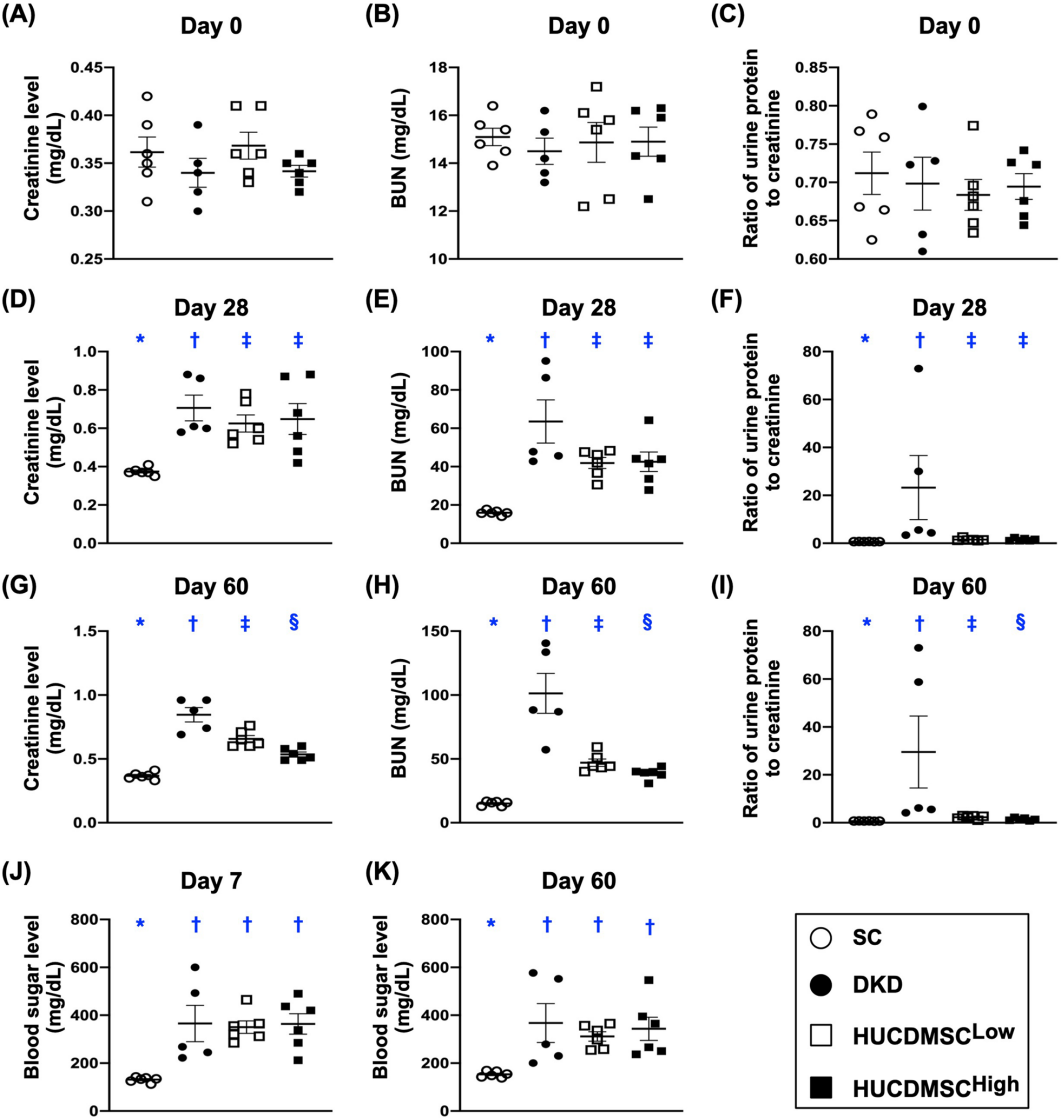
Time courses of circulating levels of BUN and creatinine and the ratio of urine protein to urine creatinine. **A** By day 0, circulating level of creatinine, *p* > 0.5. **B** By day 0, circulating level of blood urea nitrogen (BUN), *p* > 0.5. **C** By day 0, the ratio of urine protein to urine creatinine (Ra-Up/Uc), *p* > 0.5. **D** By day 28, circulating level of creatinine, * versus other groups with different symbols (†, ‡), *p* < 0.001. **E** By day 28, circulating level of BUN, * versus other groups with different symbols (†, ‡), *p* < 0.001. **F** By day 28, the Ra-Up/Uc, * versus other groups with different symbols (†, ‡), *p* < 0.001. **G** By day 60, the circulating level of creatinine, * versus other groups with different symbols (†, ‡, §), *p* < 0.0001. **H** By day 60, circulating level of BUN, * versus other groups with different symbols (†, ‡, §), *p* < 0.0001. **I** By day 60, the Ra-Up/Uc, * versus other groups with different symbols (†, ‡, §), *p* < 0.0001. **J** By day 7, the blood sugar level (SC vs. all DKD), * versus †, *p* < 0.0001. **K** By day 60, the blood sugar level (SC vs. all DKD), * versus †, *p* < 0.001. All statistical analyses were performed by one-way ANOVA, followed by Bonferroni multiple comparison post hoc test (*n* = 6 for each group). Symbols (*, †, ‡, §) indicate significance (at 0.05 level). SC = sham-operated control; DKD = diabetic kidney disease; HUCDMSC^Low^ = human umbilical cord-derived mesenchymal stem cell of lower dose (2.1 × 10^5^ cells); HUCDMSC^High^ = human umbilical cord-derived mesenchymal stem cell of higher dose (6.3 × 10^5^ cells)

The baseline circulatory levels of BUN and creatinine and the ratio of urine protein to urine creatinine (Ra-Up/Uc) did not differ among the four groups (Fig. 1). However, by day 28, these three parameters were significantly lower in group 1 (i.e., SC) than in groups 2 (DKD), 3 (DKD + lower-dose HUCDMSCs) and 4 (DKD + higher-dose HUCDMSCs) (Fig. 1). On the other hand, these three parameters did not differ among groups 2 to 4 at this time point (Fig. 1). Additionally, by day 60 (i.e., at the end of study), these three parameters were lowest in group 1, highest in group 2 and significantly higher in group 3 than in group 4, implicating that higher dose of HUCDMSCs could be superior to the lower counterpart on protecting the residual renal function in setting of DKD (Fig. 1).

At baseline, the blood sugar did not differ among the four groups (Fig. 1). However, by day 7 after STZ administration, this parameter was significantly lower in group 1 than in groups 2 to 4, but it showed no difference among groups 2 to 4 (Fig. 1). By day 60 after DKD induction, this parameter was significantly lower in group 1 than in groups 2 to 4, whereas blood sugar did not differ among these latter three groups (Fig. 1).

### Identification of kidney injury score and fibrotic area in kidney parenchyma by day 60 after DKD induction

**Fig. 2:**
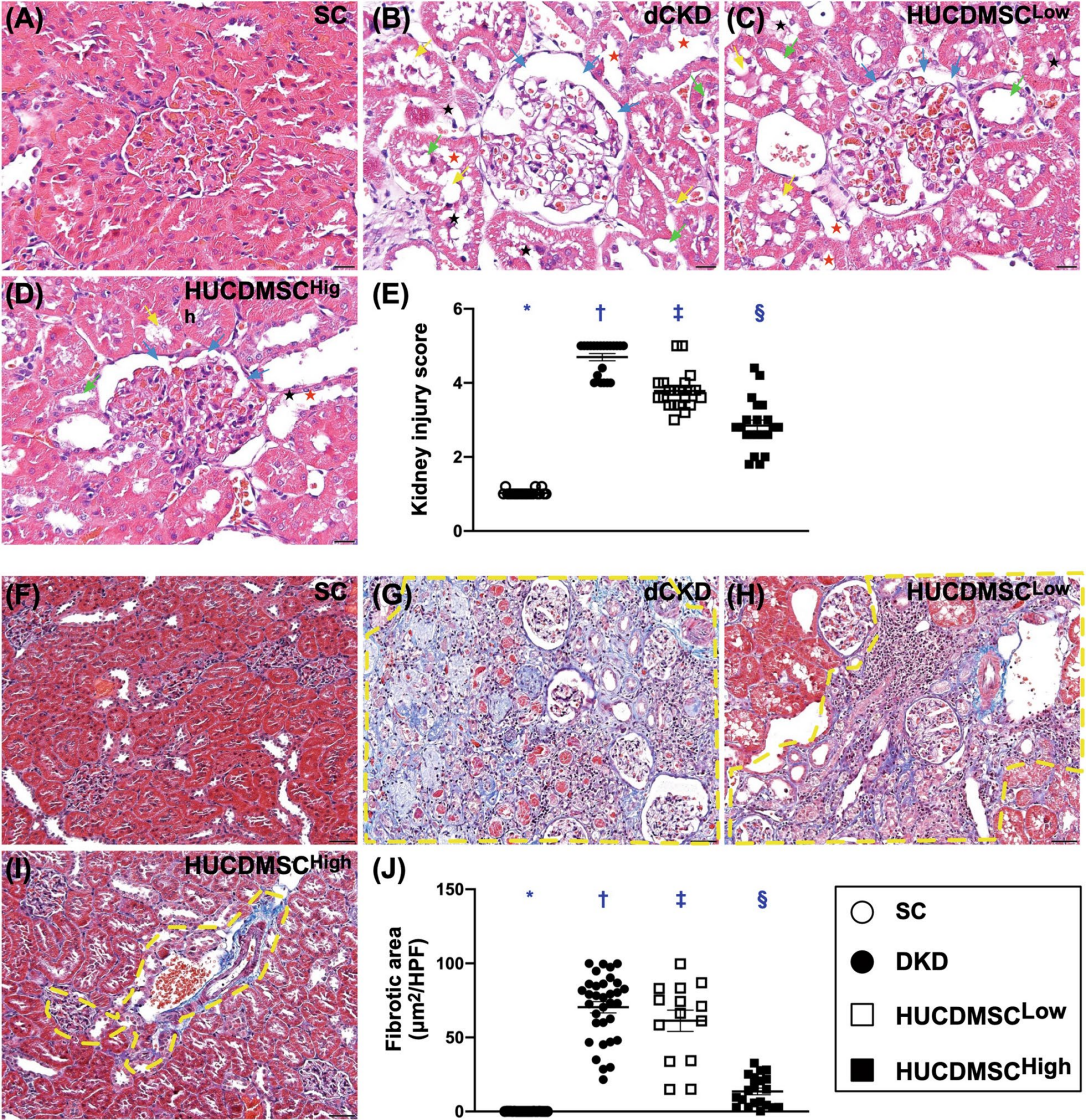
Quantitative assessment of the kidney injury score and fibrotic area in kidney parenchyma by day 60 after DKD induction. **A**–**D** Microscopic examination (200x; H&E stain) demonstrating significantly higher loss of brush border in renal tubules (yellow arrows), tubular necrosis (green arrows), tubular dilatation (red asterisk), protein cast formation (black asterisk) and dilatation of Bowman’s capsule (blue arrows) in DKD group than in other groups. **E** Analytical result of kidney injury score, * versus other groups with different symbols (†, ‡, §), *p* < 0.0001. **F**–**I** Demonstrating the microscopic finding [(200x) for identification of fibrotic area (blue color) (red dotted lines)]. **J** Analytical result of fibrotic area, * versus other groups with different symbols (†, ‡, §), *p* < 0.0001. All statistical analyses were performed by one-way ANOVA, followed by Bonferroni multiple comparison post hoc test (*n* = 6 for each group). Symbols (*, †, ‡, §) indicate significance (at 0.05 level). SC = sham-operated control; DKD = diabetic kidney disease; HUCDMSC^Low^ = human umbilical cord-derived mesenchymal stem cell of lower dose (2.1 × 10^5^ cells); HUCDMSC^High^ = human umbilical cord-derived mesenchymal stem cell of higher dose (6.3 × 10^5^ cells)

To evaluate the therapeutic impact of HUCDMSCs on protecting the kidney architecture, the H.E. stain and IHC stain were utilized in the present study. As we expected, the kidney injury score was lowest in group 1, highest in group 2 and significantly higher in group 3 than in group 4 (Fig. 2). Additionally, the Mason’s trichrome stain demonstrated that the fibrotic area in the kidney parenchyma exhibited an identical pattern of kidney injury score among the four groups, suggesting that the HUCDMSCs therapy would protect the integrity of kidney parenchyma (i.e., microstructural integrity) (Fig. 2). Of importance was that there was no tumorigenesis identified by the histopathological analysis.

### HUCDMSCs therapy preserved the integrity of ultrastructural glomeruli and attenuated the renal tubular injury by day 60 after DKD induction

**Fig. 3:**
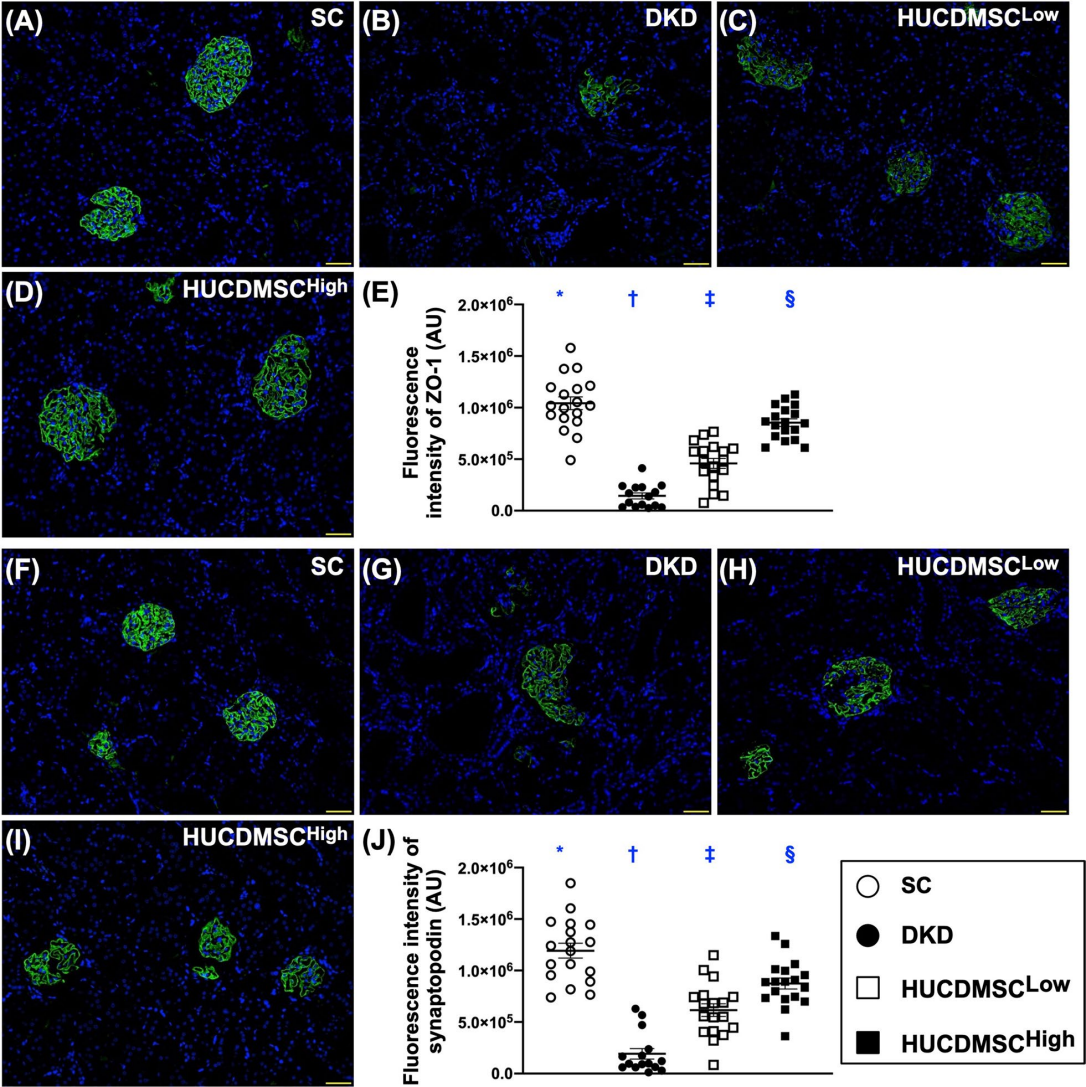
HUCDMSCs therapy preserved the podocyte components of glomerulus by day 60 after DKD induction. **A**–**D** The immunofluorescent (IF) microscopic finding (200x) for identification of fluorescent intensity of zonula occludens-1 (ZO-1) (green color). **E** Analytical result of mean fluorescent intensity ZO-1, * versus other groups with different symbols (†, ‡, §), *p* < 0.0001. Scale bar in right lower corner represents 50 µm. **F**–**I** The IF microscopic finding (200x) for identification of fluorescent intensity of synaptopodin (green color). **J** Analytical result of mean fluorescent intensity of synaptopodin, * versus other groups with different symbols (†, ‡, §), *p* < 0.0001. Scale bar in right lower corner represents 20 µm. All statistical analyses were performed by one-way ANOVA, followed by Bonferroni multiple comparison post hoc test (*n* = 6 for each group). Symbols (*, †, ‡, §) indicate significance (at 0.05 level). SC = sham-operated control; DKD = diabetic kidney disease; HUCDMSC^Low^ = human umbilical cord-derived mesenchymal stem cell of lower dose (2.1 × 10^5^ cells); HUCDMSC^High^ = human umbilical cord-derived mesenchymal stem cell of higher dose (6.3 × 10^5^ cells)

To explore deeper regarding the ultrastructural integrity of glomeruli in DKD rodent after receiving HUCDMSCs therapy, IF and IHC microscopic examinations were conducted. The result showed that the fluorescent expressions of ZO-1 and synaptopodin (Fig. 3), two components of podocytes, were highest in group 1, lowest in group 2 and significantly lower in group 3 than in group 4.

**Fig. 4:**
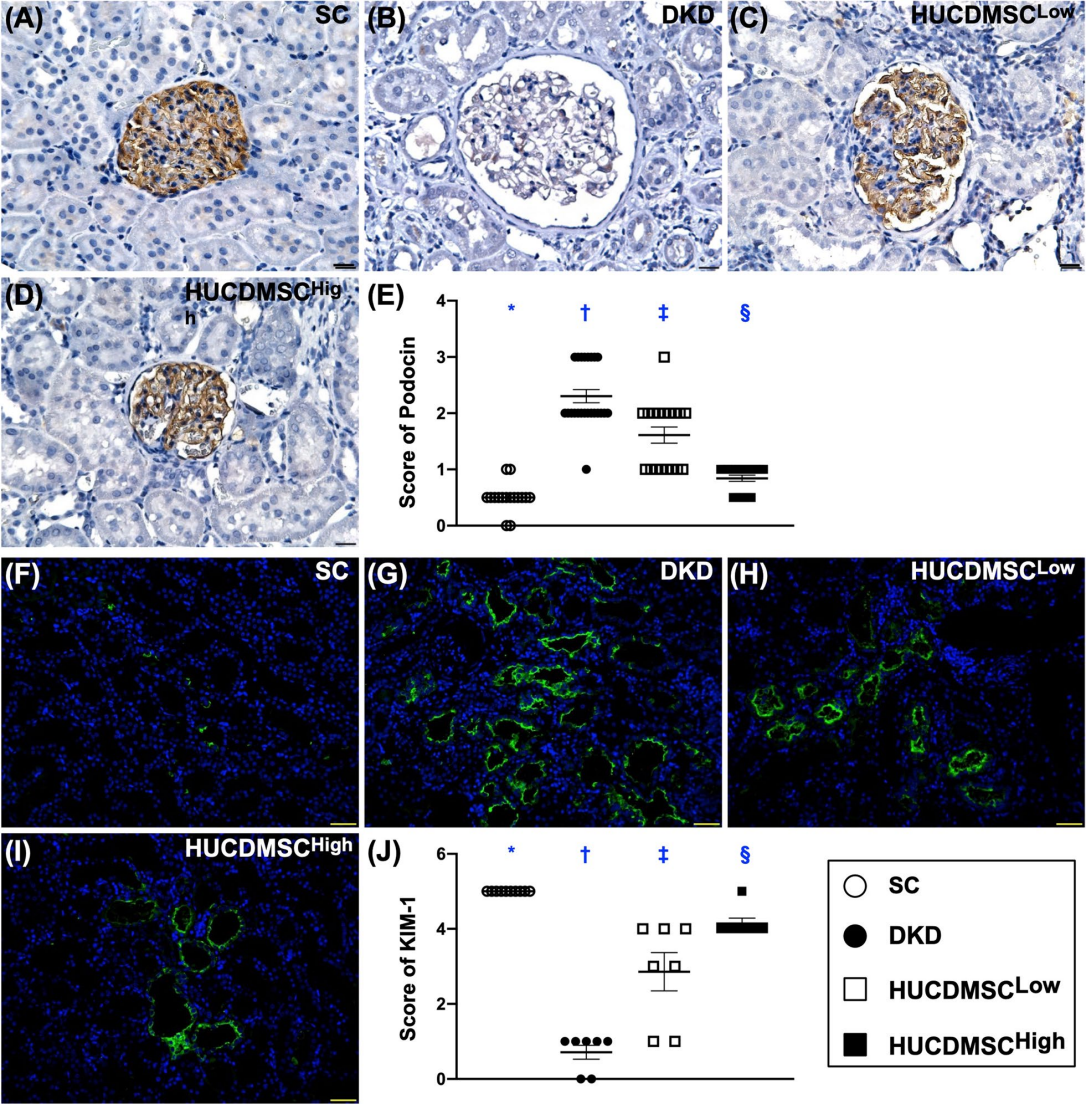
HUCDMSCs therapy preserved the integrity of component of slip diaphragm in glomeruli and attenuated the renal tubular injury marker by day 60 after DKD induction. **A**–**D** The microscopic finding (400x) of immunohistochemical (IHC) stain for the identification of cellular expression of podocin (gray color). **E** Analytical result of expression of podocin, * versus other groups with different symbols (†, ‡, §), *p* < 0.0001. **F**–**I** Showing the IF microscopic finding (400x) for the identification of kidney injury molecule (KIM)-1 (green color). **J** Analytical result of expression of KIM-1, * versus other groups with different symbols (†, ‡, §), *p* < 0.0001. Scale bar in right lower corner represents 20 µm. All statistical analyses were performed by one-way ANOVA, followed by Bonferroni multiple comparison post hoc test (*n* = 6 for each group). Symbols (*, †, ‡, §) indicate significance (at 0.05 level). SC = sham-operated control; DKD = diabetic kidney disease; HUCDMSC^Low^ = human umbilical cord-derived mesenchymal stem cell of lower dose (2.1 × 10^5^ cells); HUCDMSC^High^ = human umbilical cord-derived mesenchymal stem cell of higher dose (6.3 × 10^5^ cells)

Additionally, the cellular expression of podocin (Fig. 4), another podocyte component, displayed an identical pattern, whereas the cellular expression of KIM-1, an indicator of renal tubular damage, displayed an opposite pattern of ZO-1 among the four groups, suggesting that HUCDMSCs therapy protected the kidney ultrastructure. These findings could, at least in part, explain why the proteinuria was remarkably reduced in DKD animals after receiving HUCDMSCs treatment.

### HUCDMSCs therapy suppressed apoptotic, fibrotic and autophagic biomarkers in kidney parenchyma by day 60 after DKD induction

**Fig. 5:**
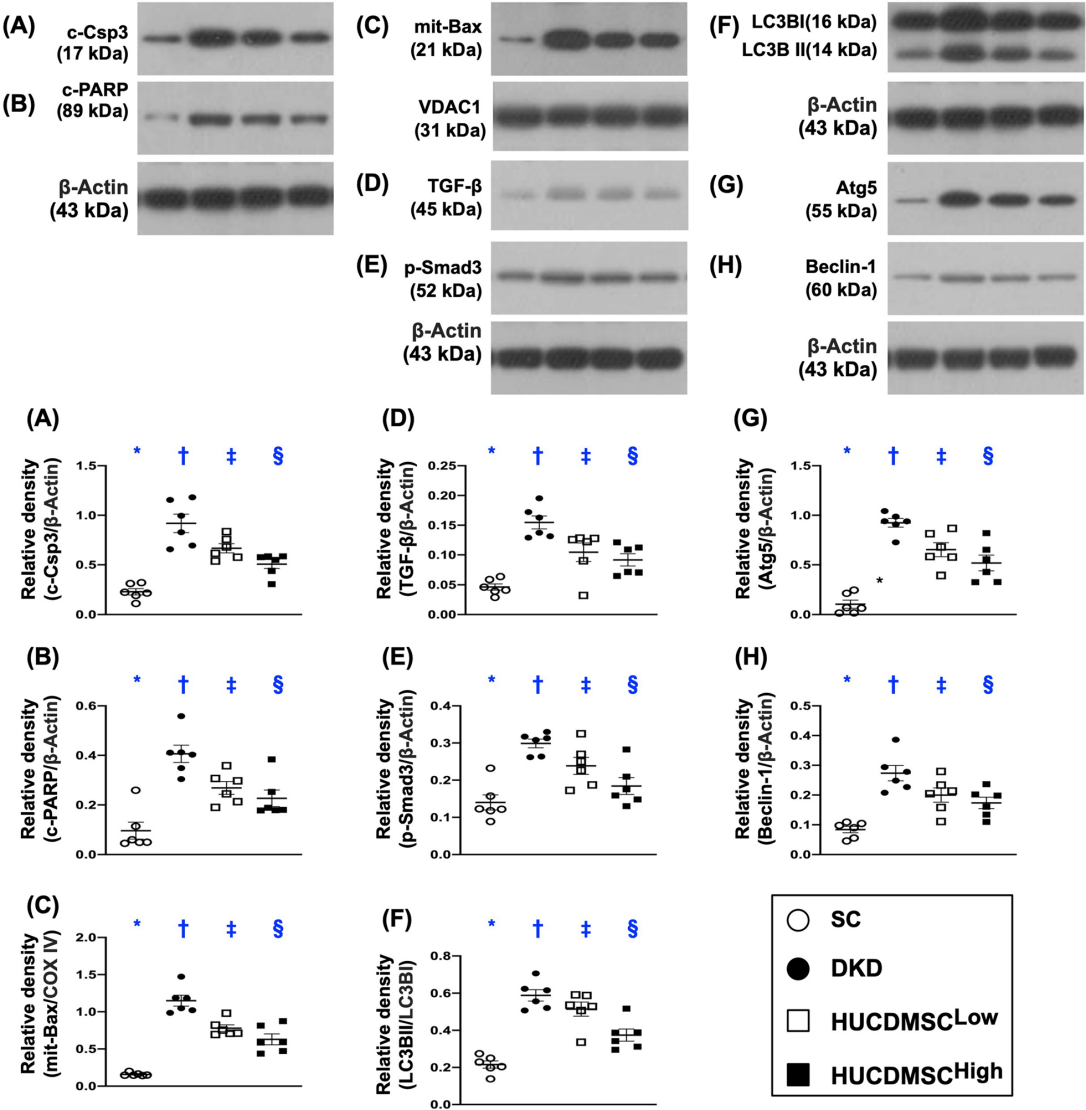
HUCDMSCs therapy suppressed apoptotic, fibrotic and autophagic biomarkers in kidney parenchyma by day 60 after DKD induction. **A** Protein expression of cleaved caspase-3 (c-Csp3), * versus other groups with different symbols (†, ‡, §), *p* < 0.0001. **B** Protein expression of cleaved poly (ADP-ribose) polymerase (c-PARP), * versus other groups with different symbols (†, ‡, §), *p* < 0.0001. **C** Protein expression of mitochondrial Bax (mit-Bax), * versus other groups with different symbols (†, ‡, §), *p* < 0.0001. **D** Protein expression of transforming growth factor (TGF)-ß, * versus other groups with different symbols (†, ‡, §), *p* < 0.0001. **E** Protein expression of phosphorylated (p)-Smad3, * versus other groups with different symbols (†, ‡, §), *p* < 0.0001. **F** Protein expression of the ratio of LC3B-II to LC3B-I, * versus other groups with different symbols (†, ‡, §), *p* < 0.0001. **G** Protein expression of Atg5, * versus other groups with different symbols (†, ‡, §), *p* < 0.0001. **H** Protein expression of beclin-1, * versus other groups with different symbols (†, ‡, §), *p* < 0.0001. All statistical analyses were performed by one-way ANOVA, followed by Bonferroni multiple comparison post hoc test (*n* = 6 for each group). Symbols (*, †, ‡, §) indicate significance (at 0.05 level). SC = sham-operated control; DKD = diabetic kidney disease; HUCDMSC^Low^ = human umbilical cord-derived mesenchymal stem cell of lower dose (2.1 × 10^5^ cells); HUCDMSC^High^ = human umbilical cord-derived mesenchymal stem cell of higher dose (6.3 × 10^5^ cells)

To investigate the severity of apoptosis, fibrosis and autophagy in the DKD kidney, the Western blot analysis was utilized in the present study. The result showed that the protein expressions of cleaved caspase-3, cleaved PARP and mitochondrial-Bax, three indices of apoptosis, were lowest in group 1, highest in group 2 and significantly higher in group 3 than in group 4 (Fig. 5). Additionally, the protein expressions of TGF-ß and p-Smad3, two indicators of fibrosis, and the ratio of LC3B-II to LC3B-I, Atg5 and beclin-1, three indicators of autophagy, exhibited an identical pattern of apoptosis among the four groups (Fig. 5).

### HUCDMSCs therapy reduced oxidative stress and mitochondrial/DNA-damaged markers in kidney parenchyma by day 60 after DKD induction

**Fig. 6:**
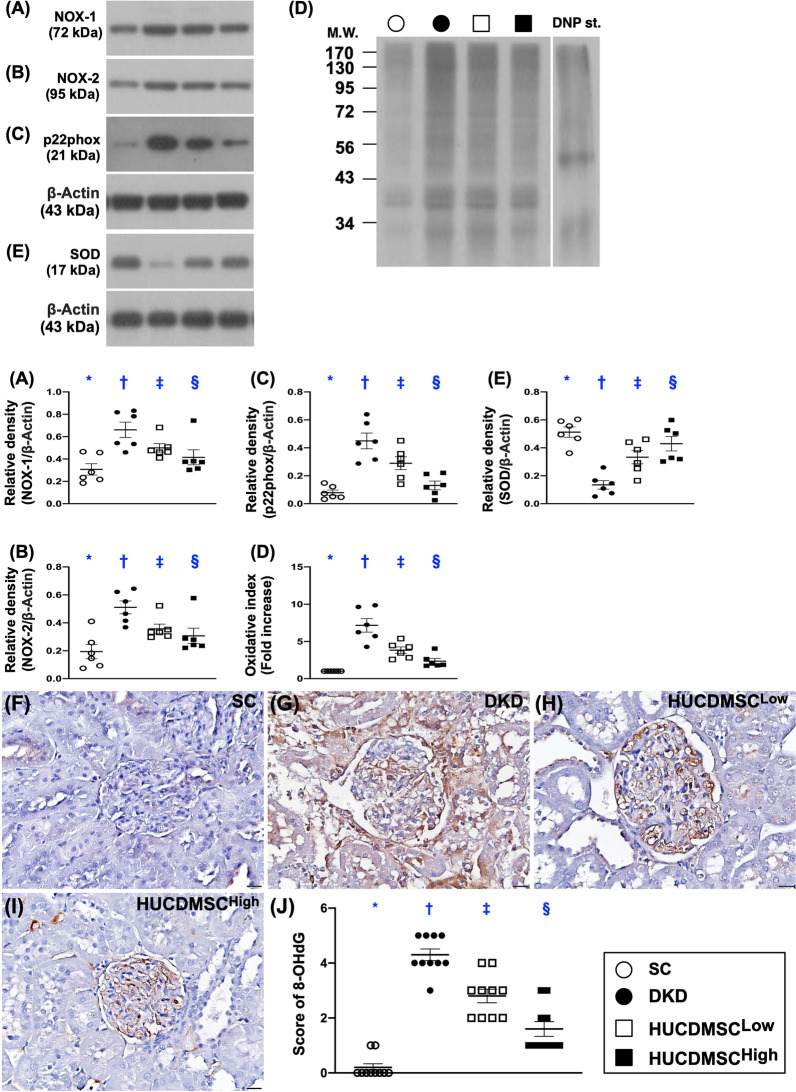
HUCDMSCs therapy reduced oxidative stress markers in kidney parenchyma by day 60 after DKD induction. **A** Protein expression of NOX-1, * versus other groups with different symbols (†, ‡, §), *p* < 0.0001. **B** Protein expression of NOX-2, * versus other groups with different symbols (†, ‡, §), *p* < 0.0001. **C** Protein expression of p22phox, * versus other groups with different symbols (†, ‡, §), *p* < 0.0001. **D** The oxidized protein expression, * versus other groups with different symbols (†, ‡, §), *p* < 0.0001, *p* < 0.0001 (Note: the left and right lanes shown on the upper panel represent protein molecular weight marker and control oxidized molecular protein standard, respectively). M.W. = molecular weight; DNP = 1–3 dinitrophenylhydrazone. **E** Protein expression of superoxide dismutase (SOD), * versus other groups with different symbols (†, ‡, §), *p* < 0.0001, *p* < 0.0001. **F**–**I** The microscopic finding (200x) for identification of cellular expression of 8-hydroxy-2' -deoxyguanosine (8-OHdG) (gray color). **J** Analytical result of expression of 8-OHdG, * versus other groups with different symbols (†, ‡, §), *p* < 0.0001. All statistical analyses were performed by one-way ANOVA, followed by Bonferroni multiple comparison post hoc test (*n* = 6 for each group). Symbols (*, †, ‡, §) indicate significance (at 0.05 level). SC = sham-operated control; DKD = diabetic kidney disease; HUCDMSC^Low^ = human umbilical cord-derived mesenchymal stem cell of lower dose (2.1 × 10^5^ cells); HUCDMSC^High^ = human umbilical cord-derived mesenchymal stem cell of higher dose (6.3 × 10^5^ cells)

**Fig. 7:**
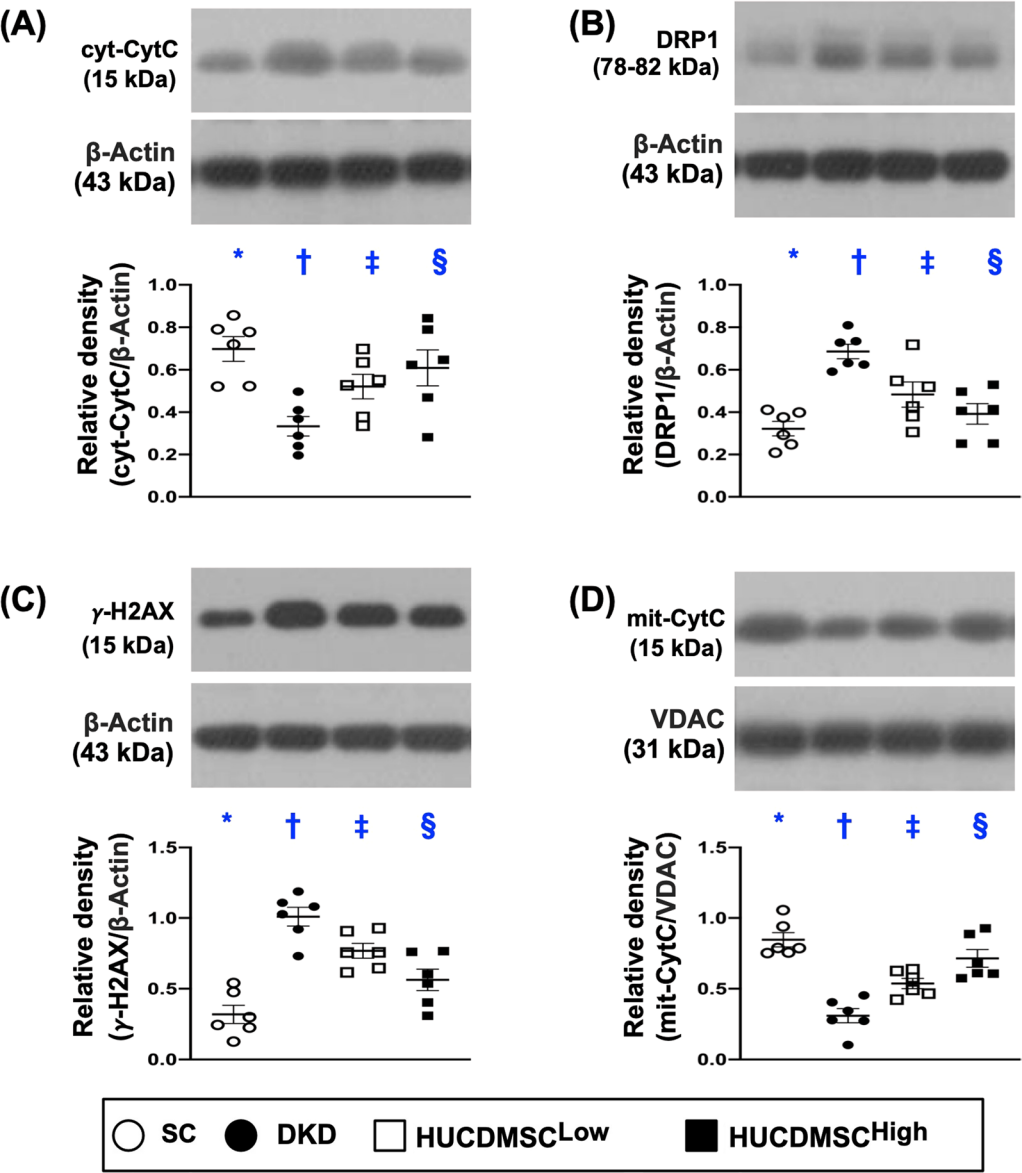
HUCDMSCs therapy reduced mitochondrial and DNA-damaged biomarkers in kidney parenchyma by day 60 after DKD induction. **A** Protein expression of cytosolic cytochrome C (cyt-CytC), * versus other groups with different symbols (†, ‡, §), *p* < 0.0001. **B** Protein expression of DRP1, * versus other groups with different symbols (†, ‡, §), *p* < 0.0001. **C** Protein expression of γ-H2AX, * versus other groups with different symbols (†, ‡, §), *p* < 0.0001. **D** Protein expression of mitochondrial cytochrome C (mit-CytC), * versus other groups with different symbols (†, ‡, §), *p* < 0.0001. All statistical analyses were performed by one-way ANOVA, followed by Bonferroni multiple comparison post hoc test (*n* = 6 for each group). Symbols (*, †, ‡, §) indicate significance (at 0.05 level). SC = sham-operated control; DKD = diabetic kidney disease; HUCDMSC^Low^ = human umbilical cord-derived mesenchymal stem cell of lower dose (2.1 × 10^5^ cells); HUCDMSC^High^ = human umbilical cord-derived mesenchymal stem cell of higher dose (6.3 × 10^5^ cells)

Western blot again was utilized for analysis of protein levels of oxidative stress and mitochondrial/DNA-damaged biomarkers. The result showed that the protein expressions of NOX-1, NOX-2, oxidized protein, p22phox and the cellular level of 8-OHdG, five indices of oxidative stress, were lowest in group 1, highest in group 2 and significantly higher in group 3 than in group 4 (Fig. 6). On the other hand, the protein expression of SOD, an indicator of antioxidant, exhibited an opposite pattern of oxidative stress among the groups. Additionally, the protein expressions of cytosolic cytochrome C and DRP1 (Fig. 7), two indicators of mitochondrial damaged markers, and protein expression of γ-H2AX (Fig. 7), an indicator of DNA-damaged biomarker, displayed an identical pattern, whereas the protein expression of mitochondrial cytochrome C (Fig. 7), an indicator of mitochondrial integrity, exhibited an opposite pattern of oxidative stress among the four groups.

### HUCDMSCs therapy downregulated inflammation and upregulated angiogenesis factors in kidney parenchyma by day 60 after DKD induction

**Fig. 8:**
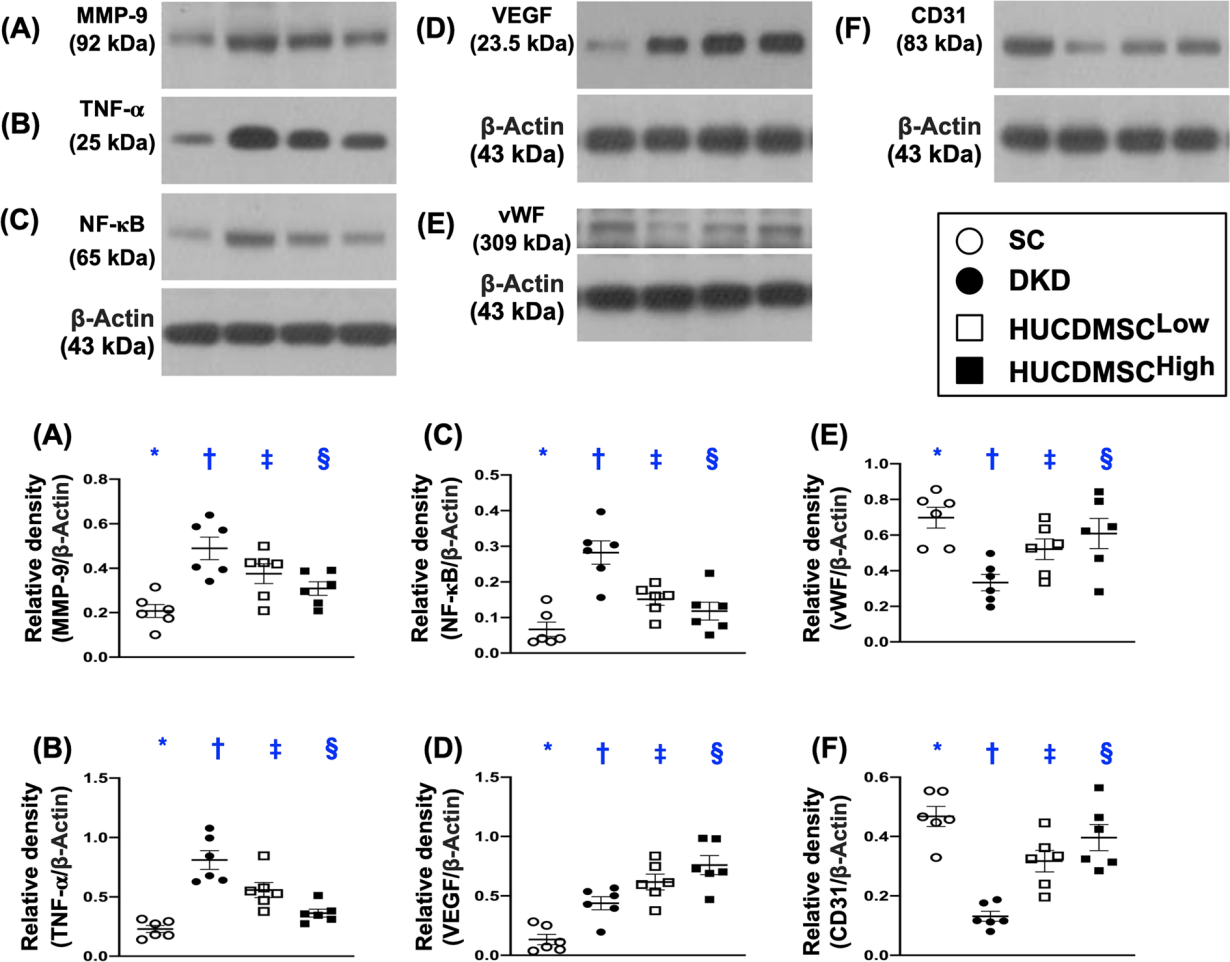
HUCDMSCs therapy suppressed inflammation and augmented angiogenesis factors in kidney parenchyma by day 60 after DKD induction. **A** Protein expression of matrix metalloproteinase (MMP)-9, * versus other groups with different symbols (†, ‡), *p* < 0.0001. **B** Protein expression of tumor necrosis factor (TNF)-α, * versus other groups with different symbols (†, ‡), *p* < 0.0001. **C** Protein expression of phosphorylated nuclear factor (p-NF)-κB, * versus other groups with different symbols (†, ‡, §), *p* < 0.0001. **D** Protein expression of vascular endothelial growth factor (VEGF), * versus other groups with different symbols (†, ‡, §), *p* < 0.0001. **E** Protein expression of von Willebrand factor (vWF), * versus other groups with different symbols (†, ‡), *p* < 0.0001. **F** Protein expression of CD31, * versus other groups with different symbols (†, ‡), *p* < 0.0001. All statistical analyses were performed by one-way ANOVA, followed by Bonferroni multiple comparison post hoc test (*n* = 6 for each group). Symbols (*, †, ‡, §) indicate significance (at 0.05 level). SC = sham-operated control; DKD = diabetic kidney disease; HUCDMSC^Low^ = human umbilical cord-derived mesenchymal stem cell of lower dose (2.1 × 10^5^ cells); HUCDMSC^High^ = human umbilical cord-derived mesenchymal stem cell of higher dose (6.3 × 10^5^ cells)

Finally, we utilized Western blot once again to elucidate the inflammation and angiogenesis biomarkers. As expected, the protein expressions of MMP-9, TNF-α and p-NF-κB, three indices of inflammatory reaction, were lowest in group 1, highest in group 2 and significantly higher in group 3 than in group 4 (Fig. 8). On the other hand, the protein expressions of CD31 and vWF, two indicators of endothelial functional integrity and angiogenesis as well as the protein expression of VEGF, an angiogenesis biomarker, exhibited an opposite pattern of inflammation among the four groups (Fig. 8).

### HUCDMSCs therapy upregulated vascularity in kidney parenchyma by day 60 after DKD induction

**Fig. 9:**
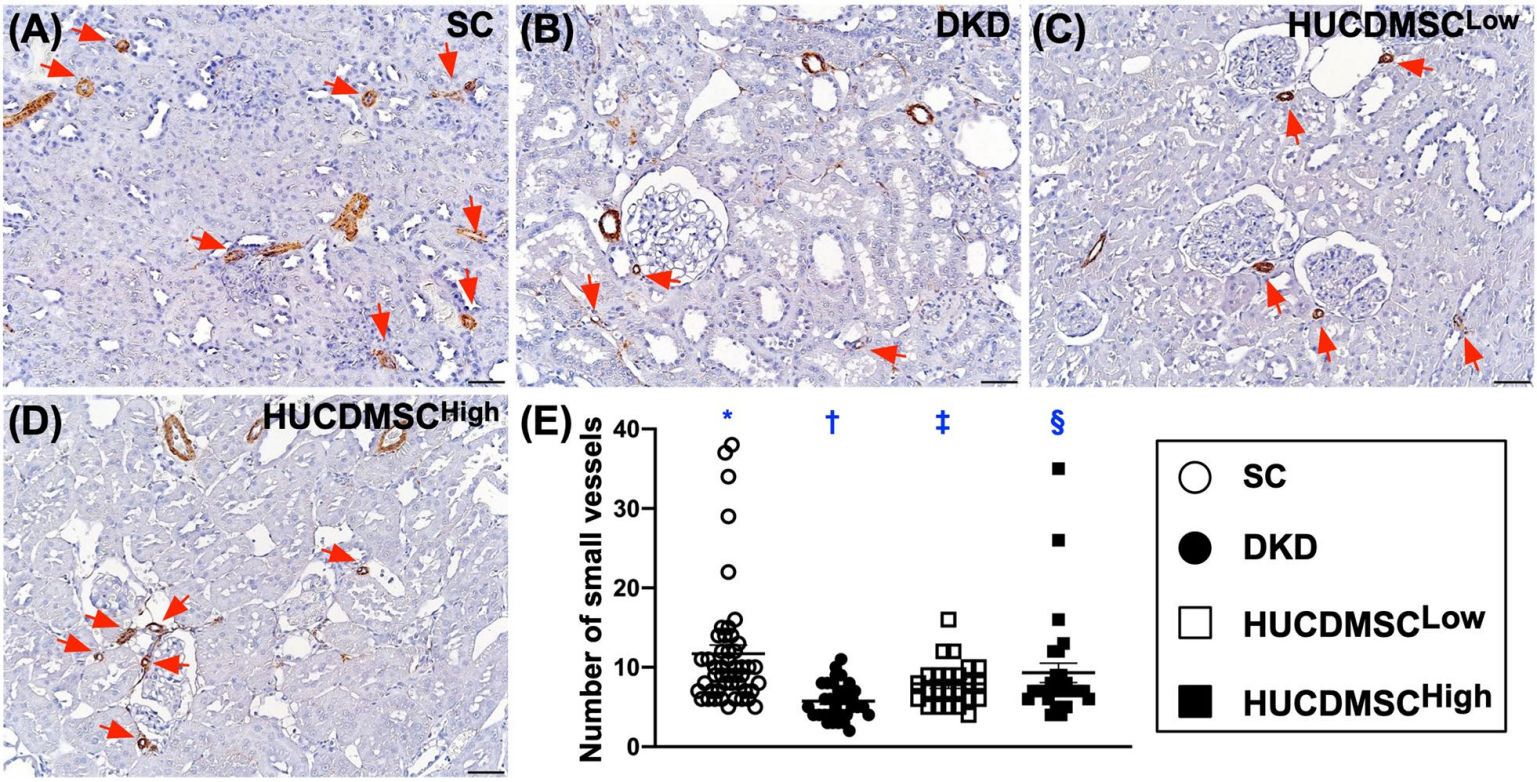
HUCDMSCs therapy enhanced the expressions of small vessel density in kidney parenchyma by day 60 after DKD induction. **A**–**D** Microscopic finding (100x) of alpha-smooth muscle actin (α-SMA) stain for identification of small vessels (gray color) in kidney parenchyma. **E** Analytic results of number of small vessel (≤ 25 μm), * versus other groups with different symbols (†, ‡, §), *p* < 0.0001. Scale bars in right lower corner represent 100 µm. All statistical analyses were performed by one-way ANOVA, followed by Bonferroni multiple comparison post hoc test (*n* = 6 for each group). Symbols (*, †, ‡, §) indicate significance (at 0.05 level). SC = sham-operated control; DKD = diabetic kidney disease; HUCDMSC^Low^ = human umbilical cord-derived mesenchymal stem cell of lower dose (2.1 × 10^5^ cells); HUCDMSC^High^ = human umbilical cord-derived mesenchymal stem cell of higher dose (6.3 × 10^5^ cells)

To verify whether HUCDMSCs therapy could also enhance vascularity in kidney parenchyma, we perform α-SMA staining for identification of small vessels (i.e., ≤ 25 μm). The result demonstrated that the number of small vessels was the lowest in group 2, highest in group 1 and significantly higher in group 4 than in group 3 (Fig. 9). Our finding not only implicated that HUCDMSCs therapy could augment angiogenesis but also proved that higher dosage of HUCDMSCs was superior to lower dosage counterpart for generation of small vessels in kidney parenchyma in DKD rat.

### The time courses of the DKD induction and treatment procedure as well as identification of the HUCDMSCs in the kidney parenchyma

**Fig. 10:**
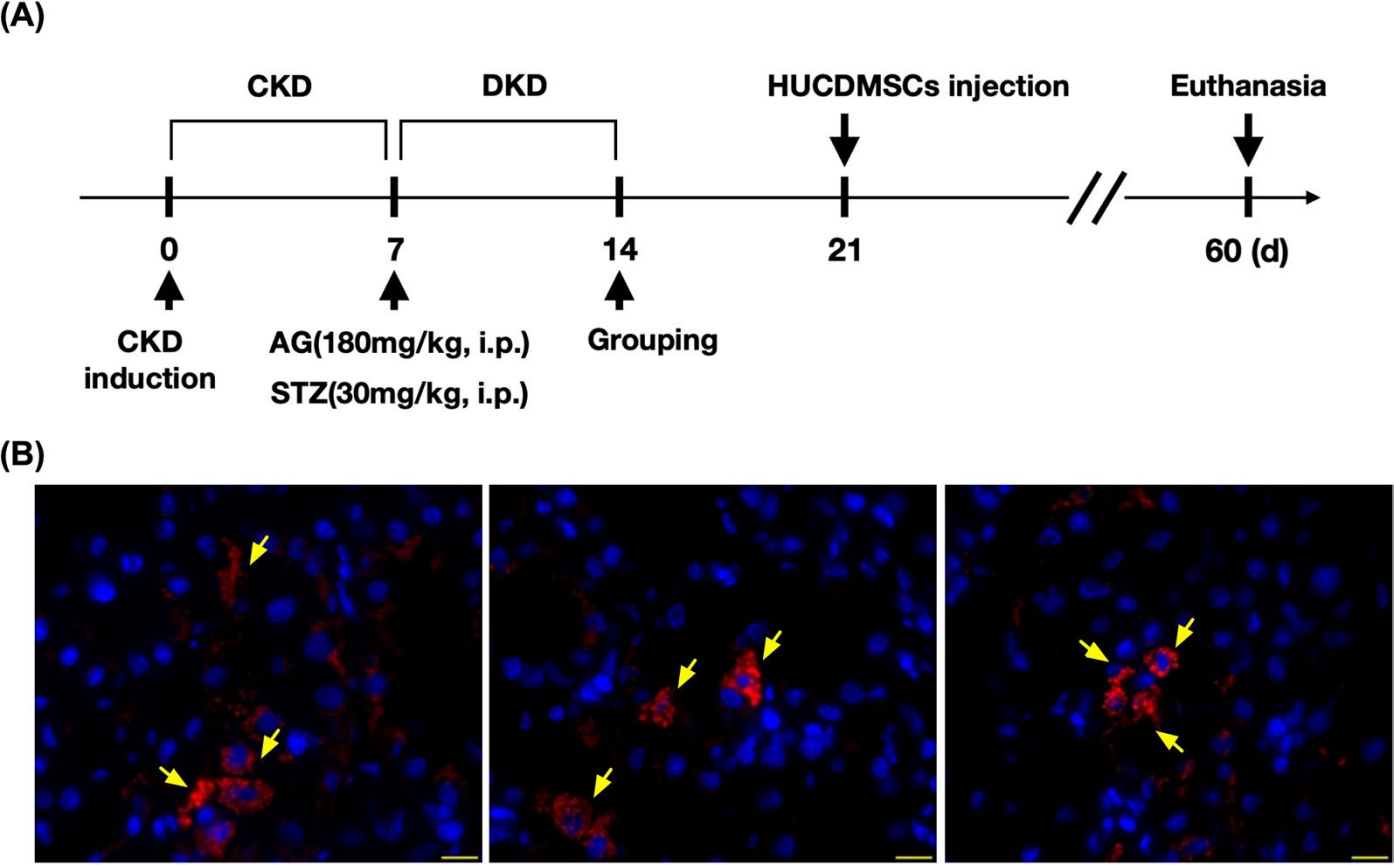
Schematic of the time courses of the DKD induction and treatment procedure as well as identification of the HUCDMSCs in the kidney parenchyma. **A** the DKD induction time points and the time interval of HUCDMSCs intrarenal arterial injection. **B** Abundant HUCDMSCs (yellow arrows) were still clearly identified in the kidney parenchyma by day 14 after intrarenal arterial cell transfusion. Note that the red color of the HUCDMSCs was clearly observed under the immunofluorescent (IF) microscopic finding (400x, scale bar over the right lower corner = 20 μM) due to the fact that these cells were stained by Qtracker™. DKD = diabetic kidney disease; HUCDMSCs = human umbilical cord-derived mesenchymal stem cells; AG = aminoguanidine; STZ = streptozotocin

The purpose of Fig. 10 was to clearly illustrate the time points of DKD induction and the timing of HUCDMSCs intrarenal arterial injection. To verify whether the transfused HUCDMSCs were still present and further integrated into the kidney parenchyma, three additional HUCDMSCs-treated animals were euthanized by day 14 after intrarenal arterial transfusion of HUCDMSCs. The IF microscopic finding demonstrated that abundant HUCDMSCs (yellow arrows, i.e., labeled by Qtracker™ 655 Cell Labeling Kit, Invitrogen, Cat. No: Q25021MP) 30 min prior to intrarenal arterial administration remained clearly identified in the kidney parenchyma by day 14 after intrarenal arterial cell transfusion, suggesting that such a procedure (i.e., by intrarenal arterial transfusion of MSCs) was safe and feasible.

## Discussion

This study which investigated the therapeutic impact of HUCDMSCs on rat DKD yielded several striking implications. First, to the rodent, the HUCDMSCs were xenogeneic MSCs. However, no immune rejection was found and there was no tumorigenesis identified in the present study. Second, HUCDMSCs therapy effectively preserved the residual renal function and markedly reduced proteinuria in DKD rats. Third, HUCDMSCs therapy significantly protected the anatomical and ultrastructural integrities of kidneys in DKD rats. Finally, HUCDMSCs therapy remarkably reduced the inflammation, oxidative stress, and mitochondrial damage in DKD rats.

Our previous studies have shown that either autologous or allogeneic MSCs therapy significantly protected the kidney and the residual renal function in CKD and in acute kidney ischemia–reperfusion (IR) injury [33, 35, 41, 43]. The most important finding was that even utilization of xenogeneic MSCs (i.e., HUCDMSCs), an effective preservation of renal function and attenuation of the proteinuria in DKD rodent were still found in the present study. Another important finding was that neither immune rejection nor tumorigenesis in kidney was found in the present study. Our findings, in addition to extending the findings of our previous studies [44–48], highlight that all races of MSCs share the common capacities of immunoprivilege and tissue regeneration as well as ensure the organs not to be damaged in settings of CKD and acute kidney IR injury.

Previous studies have clearly showed that the kidney injury score was always markedly increased in CKD setting [35, 42, 43]. Additionally, the fibrotic biomarkers were frequently identified to substantially increase in kidney parenchyma in CKD animals [35, 42, 43]. An essential finding in the present study was that the kidney injury score and the fibrosis area (i.e., histopathological findings) remarkably increased in DKD as compared with the SC group. In this way, our findings, in addition to being comparable with the findings of previous studies [35, 42, 43], at least in part, explained why the renal functional integrity (i.e., increased creatinine level and proteinuria) was deteriorated in DKD animals in comparison with SC counterparts. Of distinctively important finding was that the deteriorated renal function was remarkably reversed in lower dose and even more reversed in higher dose of HUCDMSCs treatment.

Our previous experimental studies demonstrated that the podocyte components (i.e., the ultrastructure of glomerulus) were commonly damaged not only in CKD setting [35, 42, 43] but also in the setting of acute kidney IR injury [44]. A principal finding in the present study was that those of podocyte components (i.e., ZO-1, synaptopodin and podocin) were notably downregulated, whereas the renal tubular injury biomarker (i.e., KIM-1) was markedly upregulated in DKD animals. Our findings were consistent with the findings of our previous studies [35, 42, 43]. Moreover, the inflammatory reaction, apoptosis, oxidative stress and mitochondrial/DNA-damaged and autophagic biomarkers were substantially enhanced in those of DKD animals. These findings could, once again, explain why the renal function was significantly impaired in those of DKD animals than in those of SC animals. However, these molecular perturbations were notably reversed in lower dose and more notably reversed by higher dose of HUCDMSCs therapy, resulting in improving the renal function in these animals receiving HUCDMSCs treatment. In this way, our findings suggest that intrarenal arterial transfusion of HUCDMSCs may be a novel therapeutic method to the CKD patients, especially to DKD patients whose renal function is progressively worsening and who are refractory to conventional therapy.

An issue of concern should be addressed that our previous study demonstrated that the majority of stem cells were trapped in the lung if the administration was via venous route [45]. Thus, intravenous administration of the stem cell homing to the kidney would adversely lead to a population too low when reaching destination in setting of CKD, resulting in a probably inadequate number of stem cells for tissue regeneration. One distinctive feature of this study was that even by day 14 after cell therapy (refer to Fig. 10), quite a lot of the HUCDMSCs were still identified to be present inside the kidney parenchyma. This finding might also explain why the residual renal function was more preserved in DKD animals with than without HUCDMSCs treatment.

Finally, to mimic the clinical setting of type 2 DM in the present study, we utilize aminoguanidine to protect the animals’ β-cells in islet of pancreas from being completely destroyed by STZ. Additionally, during the type 2 DM induction, no oral hypoglycemic drug (OHA) or insulin was given to the rodents. So, blood sugar by day 7 after DKD induction was 356 ± 84 mg%, highlighting a highly successful rate of DKD induction. Furthermore, the death rate of rats prior to being categorized into specific groups was 27.5%, suggesting that quite a lot of rodents were susceptibility to STZ damage and finally succumbed to hyperglycemia.

During the study period, OHA or insulin was not administered to any group of the animals following our purpose designed to find if intrarenal arterial administration of HUCMSCs would protect the residual islet cells, resulting in ameliorating the circulating level of blood sugar. However, by the end of the study period, the blood sugar level did not differ among groups 2 to 4, suggesting that such route of cell administration did not offer any benefit for protecting the β-cells in islet.

## Study limitation

This study has limitations. Frist, although the study period was 60 days, the time interval might still not long enough to allow the complications of diabetic induction in CKD setting fully emerged. Second, due to no treatment after DM induction and after animal grouping, there could be bias that only those animals resisting the hyperglycemic damage could survive in 60-day study period. Thus, the proteinuria and circulating level of creatinine might be underestimated.

## Conclusion

In conclusion, the results of the present study showed that intrarenal arterial administration of HUCDMSCs was safe and promising for preserving residual renal function and its architecture in DKD rats.

## Data Availability

The data that support the findings of this study are available from the corresponding authors upon reasonable request.

## Abbreviations

ACEI: Angiotensin-converting enzyme inhibitor
ARB: Angiotensin II type I receptor blockade
ARDS: Acute respiratory distress syndrome
BUN: Blood urine nitrogen
CKD: Chronic kidney disease
DKD: Diabetic kidney disease
DM: Diabetes mellitus
DN: Diabetic nephropathy
DRI: Direct renin inhibitor
ESRD: End-stage renal disease
HUCDMSCs: Human umbilical cord-derived mesenchymal stem cells
IRA: Intrarenal arterial
OHA: Oral hypoglycemic drug
ROS: Reactive oxygen species
STZ: Streptozotocin
